# Off-Label Use of Naltrexone in Pica and Other Compulsive Behaviors: A Report of Two Cases

**DOI:** 10.7759/cureus.65845

**Published:** 2024-07-31

**Authors:** Samira Khan, Kunal Vij, Emili Lopez

**Affiliations:** 1 Behavioral Health, West Virginia University (WVU) Berkeley Medical Center, Martinsburg, USA; 2 School of Osteopathic Medicine, West Virginia School of Osteopathic Medicine, Lewisburg, USA; 3 Behavioral Medicine and Psychiatry, West Virginia University School of Medicine, Martinsburg, USA

**Keywords:** severe pica, hair pulling, obsessive-compulsive symptoms, autism spectrum disorder (asd), low-dose naltrexone

## Abstract

Pica is known to the medical community as an eating disorder in which individuals may ingest non-food items due to a nutritional deficiency and cause unintentional physical harm to themselves. This article discusses the cases of children with pica in addition to other comorbidities such as trichotillomania, depression, autism, and anxiety. Both patients were trialed on typical first-line treatments to address pica symptoms, including antidepressants, psychotherapy, and neurology consults, which were ineffective in treating pica symptoms. The introduction of naltrexone resulted in significant improvements, including decreased pica symptoms and improvements in depression, anxiety, and overall behaviors. These effects of naltrexone were further bolstered by the effects that occurred when both patients discontinued naltrexone for some time.

## Introduction

Compulsive behaviors are repetitive behaviors that a person feels a strong desire to fulfill in response to an obsession. Obsessions are recurrent intrusive thoughts, images, or impulses that can cause distress due to the person recognizing they are interfering with daily living [1]. Some examples of obsessions include fear of contamination, concern with symmetry or preciseness, or fear of being harmed. Hence, some compulsions may include excessive cleaning or washing hands, putting excessive time into organizing objects, and potentially avoiding certain activities or behaviors out of fear. These compulsive behaviors are commonly recognized by patients as excessive and distressing, leading to anxiety and disruption to daily living [2]. Obsessive-compulsive disorder (OCD) is a common manifestation of compulsive behaviors, but these behaviors can manifest in other ways, including eating disorders and other compulsive habits.

Similarly, pica is a complicated eating disorder that is considered largely untreatable except for reversing learned behaviors through therapy and lifestyle modifications. It is characterized by the urge to swallow non-food or non-nutritious items that may be toxic or dangerous for at least one month and is not attributed to cultural rituals or at age milestones where this type of ingestion is acceptable [3]. Some examples may include the ingestion of ash, dirt, chalk, and paper. Pica has been attributed to a multitude of factors, including nutritional deficits such as iron deficiencies [4], mental health illnesses, and negative conditions and stressors during childhood.

Moreover, trichotillomania is the compulsive act of pulling one’s own hair out, most commonly pulled from the scalp, eyebrows, or eyelashes, with reports of a sense of relief when hair is pulled out. Clinically, trichotillomania is diagnosed by the observance of hair loss not attributable to medications or medical treatment [5]. Common factors include family history, skin or hair conditions, stressful situations that result in forming a coping mechanism, and other mental health disorders such as depression, anxiety, or obsessive-compulsive disorders. Common complications are mostly social and emotional distress over the lack of control. Hairballs can also form in your digestive tract if the hair is pulled, followed by ingestion [5].

Autism spectrum disorder (ASD), however, is a developmental disability with multiple plausible etiologies not yet identified, within which obsessive and compulsive behaviors can be observed. Patients typically show issues with social communication and interaction and repetitive behaviors or interests. ASD can show a wide spectrum of signs, not necessarily including the previously mentioned symptoms. The diagnosis is clinical and is typically screened at 18 months of age or younger. Treatment includes improving quality of life and developing daily functioning through activities of daily living (ADLs) and is unique to presenting symptoms in each patient through psychotherapy and medications [6]. Medical treatment can include dopaminergic/serotonergic antagonists (e.g., haloperidol, clomipramine), anxiolytics, and mood stabilizers [7]. Certain genetic factors can increase the risk of developing ASD, such as fragile X syndrome, but other risk factors include having a sibling with ASD, complications at birth, and having parents of advanced age [8].

Although pharmacological treatment is limited, antidepressants and antipsychotic medications may be helpful while monitoring the risks and benefits closely. Cognitive-behavioral therapy is a commonly used method to reverse compulsive disorders such as pica and trichotillomania. Some studies postulate that pica and other compulsive behaviors may be a form of addiction involving a disorder of the opiate pathway [9]. Serotonin and dopamine have been postulated to be largely responsible for addictive behaviors in chronic drug users. Chronic drug users commonly show altered opiate and dopamine pathways, which are known to reinforce and promote further cravings for their drug of choice [10]. A similar mechanism has been implicated in behavioral addictions. This pathway is explored and targeted medically in the treatment of multiple compulsive disorders present in our patients.

The following cases discuss the off-label use of a medication, Naltrexone, in treating two different types of compulsive behaviors in two different pediatric patients. Psychotropics are beneficial but do require a trial period with dose adjustments before the full resolution of symptoms is seen and felt. The goal of using medications with potential off-label benefits is to broaden the medical community's knowledge and allow for large-scale studies to be conducted.

## Case presentation

Case 1

**Figure 1:**
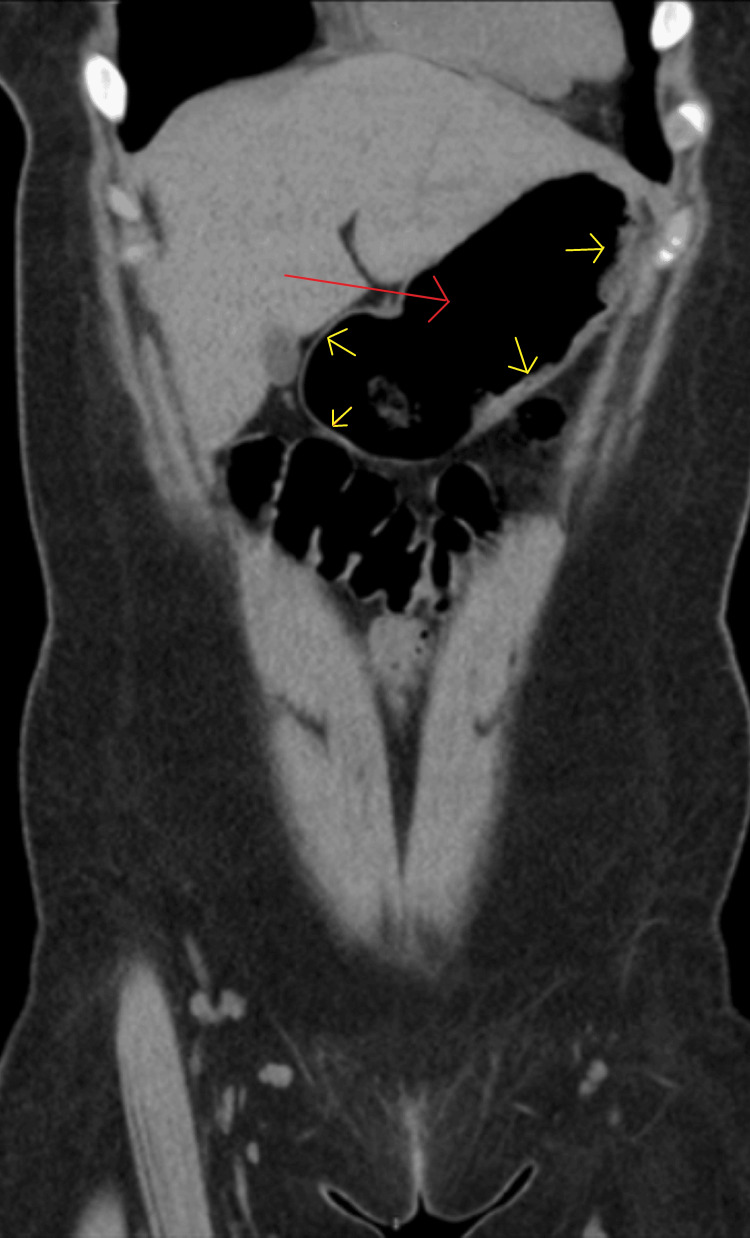
Case 1: Coronal computed tomography image depicting gastric distention (red arrow) with mild wall thickening (yellow arrows)

**Figure 2:**
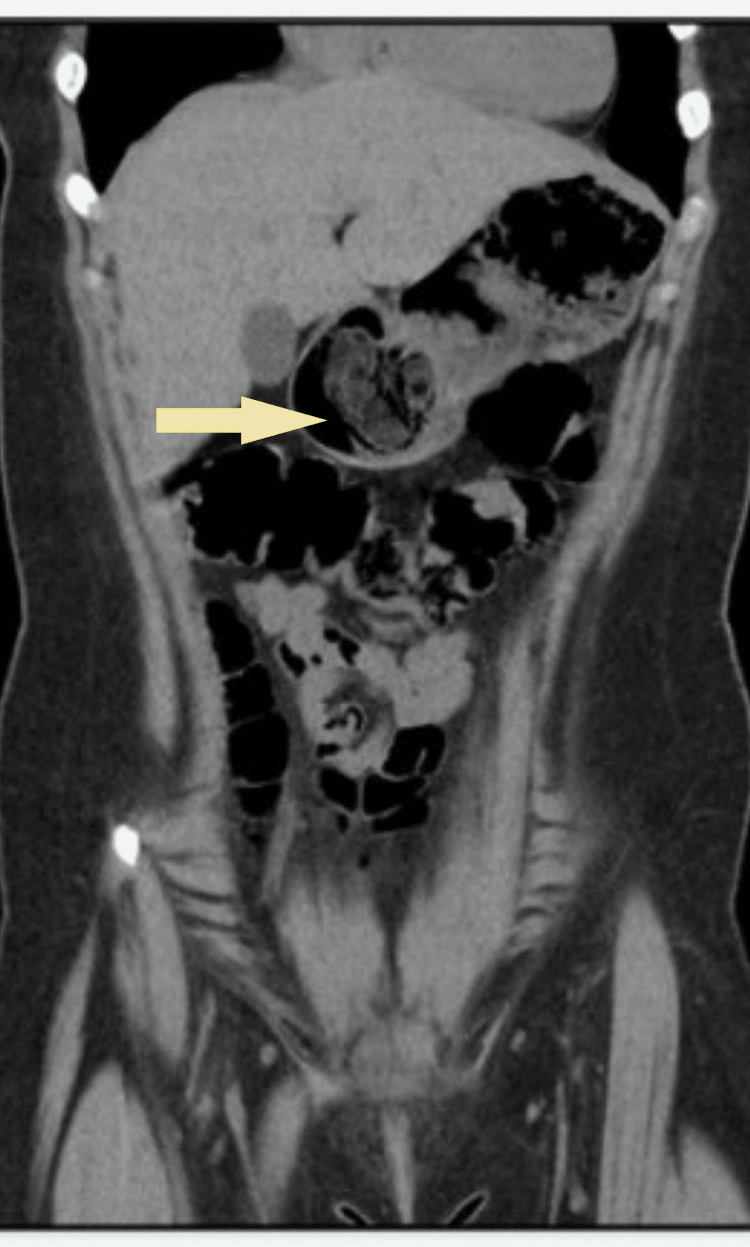
Case 1: Coronal computed tomography depicting gastric distention with ingested material (arrow)

**Figure 3:**
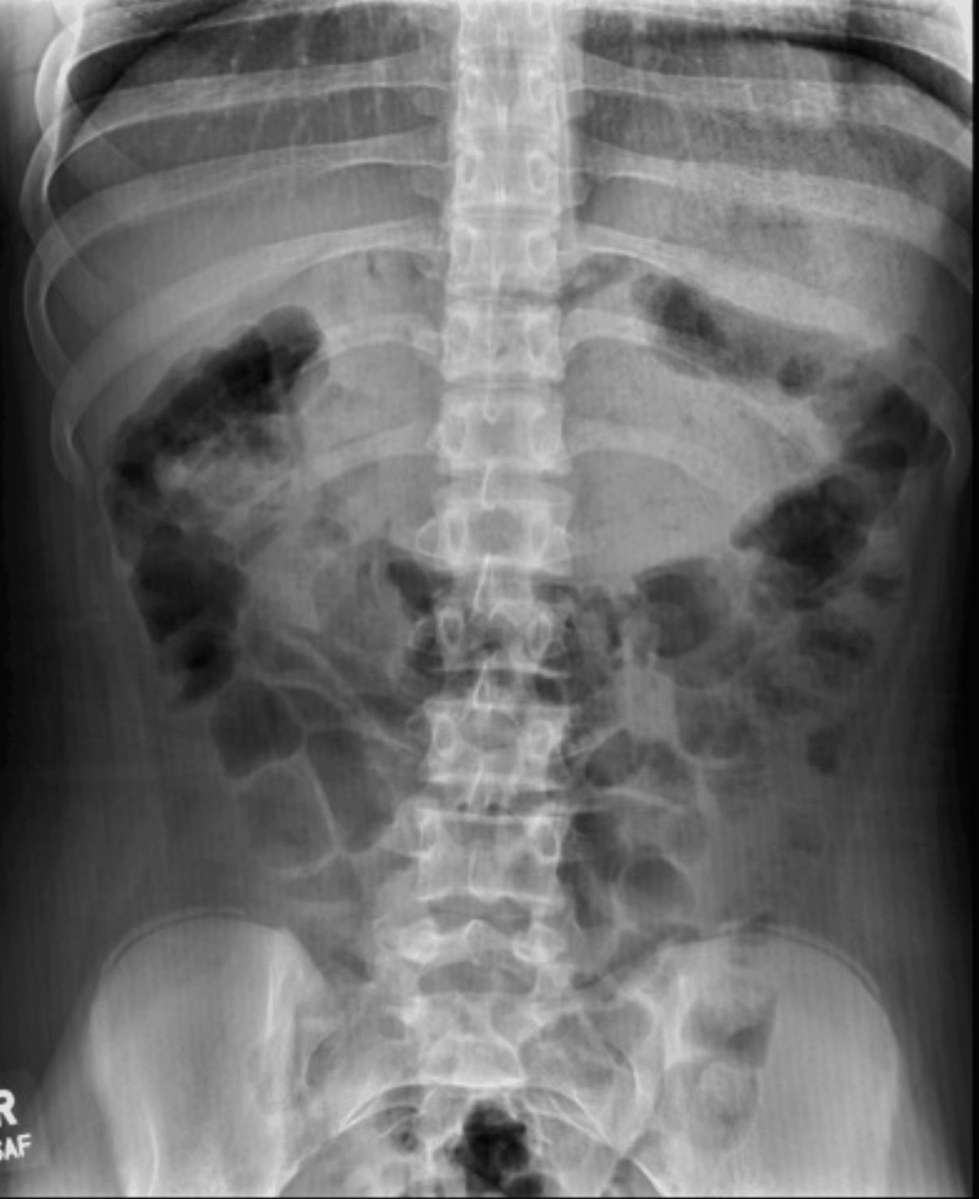
Case 1: Normal kidney and bladder X-ray

**Figure 4:**
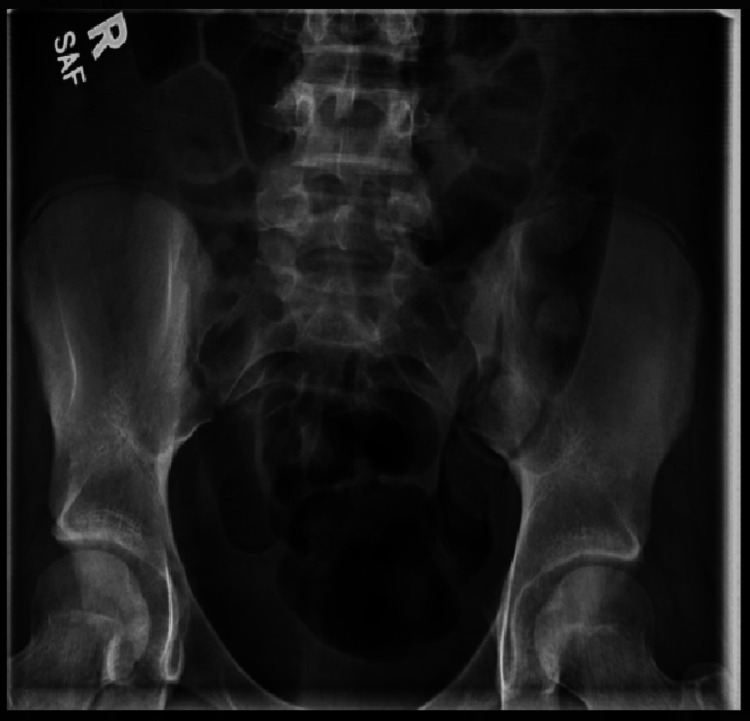
Case 1: Normal kidney, ureter, bladder X-ray

**Figure 5:**
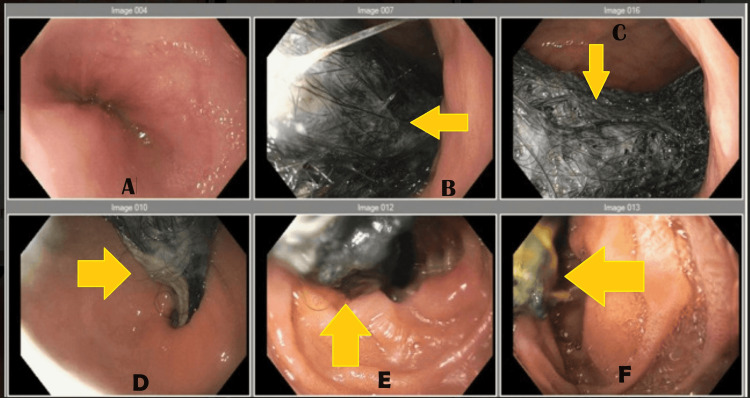
Case 1: Colonoscopy finding of a large trichobezoar indicated by arrows in panels B-F

**Figure 6:**
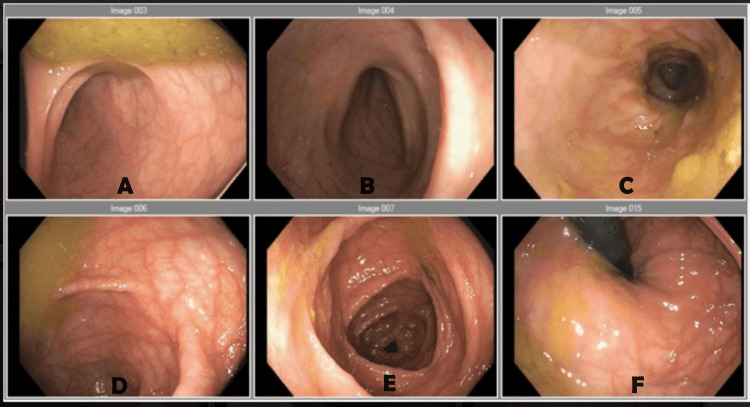
Case 1: Normal esophagogastroduodenal (EGD)

The patient is a 14-year-old girl with a past psychiatric history of pica, trichotillomania, and anxiety. Her symptoms were first noticeable when she was nine years old but progressively worsened as she got older. The patient presented to the local emergency room when she was 12 years old with abdominal pain for four days and vomiting for two days. The workup at the time included a computed tomography (CT) image scan of the abdomen (Figures 1-2), which showed presumed gastric distention with ingested material, mild wall thickening through the gastric antrum, pylorus extending to the proximal duodenum, and correlated with gastritis, duodenitis, or gastroenteritis. The rest of the workup was negative. The patient was discharged home. She returned three months later with the same complaints, and this time, she had a kidney, ureter, and bladder (KUB) X-ray (Figures 3-4), which was also negative, and the patient was discharged home. She came back two months later, complaining of cramping, epigastric pain, and vomiting. She noted her pain and vomiting were exacerbated when she ate any food item, and that led to a loss of appetite and weight loss during this period; however, the amount of weight loss was not quantified. Her workup was negative, and she was discharged home. Three weeks later, her family took her to a different hospital’s emergency room, where she also had an unremarkable workup and was discharged home. The patient saw her gastroenterologist (GI) for the first time on a video visit five months after that first ER visit. The GI provider ordered an abdominal ultrasound, colonoscopy (Figure 5), and esophagogastroduodenal (EGD) (Figure 6).

During the colonoscopy, a large trichobezoar was found, and so she was admitted to the hospital and taken to the operating room the next day for an exploratory laparotomy, gastrostomy, and removal of the bezoar.

During her post-op recovery in the hospital, she was seen by the pediatric psychiatry team and started on escitalopram. It was then that she reported that she had been pulling out her hair and chewing it for the past two years as a means to relieve anxiety and stress. She denied intentionally ingesting her hair and denied ingestion of other nonfood items, but her grandmother notes there was string and thread in the removed bezoar. She identifies her biological mother's passing a year prior as a significant stressor and school work as a stressor. Her social history was significant for the fact that she was adopted by her maternal grandparents at birth, as her biological mother struggled with heroin use and was in and out of her life. The patient was born on time and had no complications, but did require weaning from methadone.

The patient was referred for cognitive-behavioral therapy for the management of her anxiety and trichotillomania. Two months later, she reported to her pediatrician that she was no longer eating her hair. However, she continued to eat strings, and she felt she could not stop that. Regardless, she did not have any abdominal pain, nausea, or vomiting, was eating well, and had a regular bowel movement daily. However, due to increased acute life stressors, her consumption of string increased; she ate string every day to the point of having a bowel movement that was full of string and caused the toilet to clog. Her grandmother also reports that she is cutting up people's clothes to get them to eat.

Two weeks later, she was referred to psychiatry, assessed to have concerns with depression and anxiety, and restarted on escitalopram 5 mg; she had previously opted for therapy only and had self-discontinued the medication. One week later, the patient had a follow-up with her gastroenterologist for left upper quadrant (LUQ) tenderness, and a small mass was palpated in her LUQ. Abdominal US, CBC, and iron panels were ordered, and the patient was admitted to the hospital after her blood work came back positive for severe anemia with a hemoglobin of 6.9, and her grandmother noticed she looked pale, fatigued, and lethargic. She started iron supplementation a week prior to admission and stated that the iron caused her abdominal pain. A hematologist was consulted, and continued iron supplementation was recommended. GI was consulted and recommended EGD, which had normal findings except for mild to moderate chronic gastritis. A nasogastric tube was placed in the OR, and she started on the Golytely clean-out and passed clumps of string and hair. GI also recommends that stool softeners or laxatives be used once a week to get the fibers out and prevent bowel obstruction. Her abdominal pain improved, her hemoglobin stabilized, and she was discharged home. One week later, she sees her psychiatrist and reports intermittent compliance with escitalopram. She does not report adverse effects, but she does not feel much of a difference in anxiety. The patient continues to cut materials throughout the home, pull the strings out of the fabric, and eat the strings. Escitalopram was increased to 10 mg.

Six weeks later, the patient presented to the ED for left-sided abdominal pain, diarrhea, and blood in the stool. A CT abdomen showed signs of bowel wall thickening within the colon, consistent with colitis, but there was no evidence of small bowel obstruction. It also showed moderate gastric distention without concern for wall thickening. The patient was stable, workup showed that anemia had improved, and she was sent home the same day with orders for diagnostic follow-up outpatient. Three months after that, she came back to the ED for abdominal pain and reported that she had continued to eat strings. She had normal physical exam findings; KUB was normal, and she was sent home with strict return precautions. She saw her psychiatrist one month after the ED visit; escitalopram was stopped, and she started on fluoxetine 20 mg daily. Genesight testing was performed. Her grandmother states the patient has never been on escitalopram 10 mg for more than three consecutive days. According to the patient, they upset her stomach. She reports eating a lot of string on a daily basis, mostly socks; 16-18 pairs disappear per week per grandmother. On follow-up with psychiatry a month later, fluoxetine was increased to 40 mg, and naltrexone 50 mg daily was started. The GI provider ordered the upper GI to be assessed for recurrence of the bezoar, and it was normal. After being on a medication combination for a month and reporting adherence, her anxiety resolved, and her habit of eating strings significantly decreased to the point of non-existence.

Case 2

**Figure 7:**
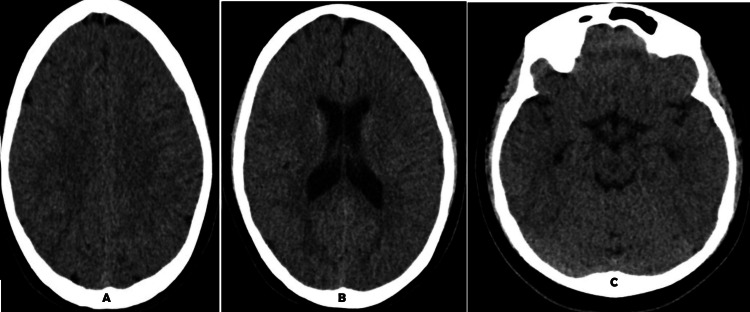
Case 2: Normal axial plane computed tomography of the brain without contrast

The patient is a 14-year-old female with a history of ASD, attention deficit and hyperactivity disorder (ADHD), and seizure like activity. The patient was 4.5 years old when she was diagnosed with ASD. There were concerns for pediatric autoimmune neuropsychiatric disorders (PANDAS) as she had multiple episodes of Strep infections, ultimately leading to her having adenoidectomy and tonsillectomy surgery. The patient started consulting a psychiatrist at age 10 for behavior concerns such as biting herself, breaking through the skin, hitting her head, and being physically and verbally aggressive toward her parents. She also appeared to be regressing in her milestones with decreasing eye contact and verbal communication. Penicillin was started for PANDAS, and a neurologist was consulted. Two weeks later, the patient presented to the ED after a fall at home and episodes of vomiting. A CT brain without contrast (Figure 7) was ordered and showed no acute findings. She was stable and discharged home the same day. The patient was then admitted to pediatric neurology for a 48-hour electroencephalography (EEG) that was negative.

At the next psychiatry appointment, two weeks after the ED visit, the patient was showing improvements in behavior. However, she was still having outbursts that were short-lived, and sleep was still an ongoing concern. Clonidine SR was increased to 0.2 mg and hydroxyzine to 50 mg. A few weeks later, the patient's Zoloft was titrated to 75 mg, hydroxyzine to 50 mg, and Clonidine SR to 0.2 mg. She was noted to have an increase in appetite and started biting herself again in the context of not getting her way. After two months, the patient was noted to be tired during the school day and was having OCD tic-like behaviors, getting stuck in a loop of repetitive movements when agitated, so Zoloft was increased to 100 mg. For about two years, her behavioral outburst was controlled before the family noted it increasing again and the patient getting agitated. Mom had concerns about possible seizures or migraine features that she was not getting addressed at the previous institution, where two EEGs were negative. She started seeing neurologists at a different institution and was diagnosed with benign intracranial hypertension and non-epileptic staring spells. After treatment initiation with acetazolamide, she was doing well. Three months later, the patient was having some rough periods secondary to school transitioning and being in a group of 13 kids with behaviors after being remote for some time. Because of this, her Zoloft was increased to 125 mg. Seven months later, the patient was noted to have started specific habits, such as biting her lips to the point of getting ulcers and scratching her skin. Hence, Zoloft was increased to 150 mg daily. Five months later, Naltrexone 50 mg once a day was started for concerns of repetitive, compulsive behaviors, biting her lips, chewing on pencils, and skin picking. At the next visit, two months later, mom reported that within the first three days of starting naltrexone, the patient’s compulsions decreased significantly and that whenever she was to skip the medication, the lip biting, skin picking, and pencil chewing would return.

## Discussion

Our patients present with several pathologies underlying compulsive behaviors, including anxiety, trichotillomania, autism, and pica. They were treated with antidepressants, including escitalopram, fluoxetine, sertraline, and other medications at varying doses. Both patients in these cases were referred to psychiatry concerning behavioral issues and psychotropic medication management.

Many factors may play a role in the development of pica, but a single theory has not been proven. Associations between adverse childhood events [11] and iron deficiency anemia have been linked to an increase in the risk of pica. A newer hypothesis suggests pica as a method of protection from toxins during periods of development; for example, clay is ingested to prevent the absorption of harmful substances [3]. This makes treatment complicated, as there is no currently known mechanism to treat it medically. For pica, diagnosis is clinically determined by the length of symptoms and mental development, and no other social, cultural, or medical factors may be attributable to the eating behaviors. The treatment focuses on halting the behavior and opting for less harmful activities. Cognitive-behavioral therapy (CBT) is commonly implemented [12]. Medically, antipsychotics may be used for pica but have not shown consistent results and are associated with more risks than benefits [3].

Medications are not primarily indicated, as the etiology of trichotillomania is also complex and largely unknown. Trichotillomania is also treated with CBT. However, first-line treatment for trichotillomania uses a different form of CBT called habit-reversal training [5]. This type of therapy focuses on replacing habits with less harmful behaviors. Similarly, autism does not have a first-line medication. Treatment focuses on improving ADLs and optimizing treatment based on the individual patient [7].

Naltrexone, a medication-assisted treatment (MAT), is a long-acting competitive opioid antagonist approved for the treatment of alcohol and opioid dependence. These drugs are known to act on dopaminergic and opiate receptors. Ethanol stimulates beta-endorphin release, leading to dopaminergic release in the nucleus accumbens, a pathway that has been extensively linked to the reward pathway [13]. Similarly, mu-opioid receptors are activated with opioid use, which further increases dopamine release in the nucleus accumbens. Both dopamine and opioid receptor activity increase cravings for the drug of choice, making these receptors an ideal target to inhibit to decrease cravings [14]. Naltrexone is commonly used as a maintenance regimen for patients willing to discontinue drug use. Naltrexone does this by inhibiting the opioid pathways responsible for propelling the reward pathway, effectively decreasing cravings for the drug of choice. Adverse effects in adult populations commonly include GI symptoms such as diarrhea and cramping. Other rarer effects, including headaches, anxiety, low energy, muscle pain, difficulty sleeping, and nausea or vomiting, have also been reported. In adults undergoing MAT with naltrexone therapy for opioid use disorder, a high dose of naltrexone is known to lead to precipitated withdrawal symptoms [15]. The effects of naltrexone on pediatric populations have not been as extensively studied, but some reports highlight that transient sedation is most commonly reported when used in patients with autism. However, the literature states that pediatric effects and efficacy are largely derived from adult studies. Pediatric doses do not exist, and the lowest dose prescribed for naltrexone is 50 mg. Developmental changes and other pharmacological properties have not been extensively studied in pediatric populations [16].

Similarly, some studies have postulated the nucleus accumbens to be responsible for eating disorders. One such study performed deep-brain stimulation targeted at the nucleus accumbens to treat anorexia nervosa [17]. Three out of seven patients had a reduction in their Eating Disorders Examination (EDE) score. Other studies suggest the potential targeting of the dopaminergic and opiate pathways for the treatment of eating disorders such as pica [18]. Naltrexone competes for opiate receptors, mainly the mu-opioid receptor, with high affinity. As our cases suggest, naltrexone is being used off-label at an increasing rate for behavioral benefits in eating disorders and other self-injurious behaviors. Naltrexone has also been shown to benefit patients with ASD in case studies and double-blind trials [19]. With these benefits and inadequate results from first-line treatments, naltrexone is used in treatment for pica and for additional benefits for anxiety, trichotillomania, depression, and autism.

Naltrexone showed significant improvements in our patients. Anxiety, hair pulling, skin biting, and ingestion of non-food objects were significantly reduced, and behaviors improved at home and school. This is the result of an optimized regimen to treat anxiety and depression, in addition to ADHD and insomnia in the second case. In both patients, we can further attribute naltrexone to improving obsessive symptoms due to the lack of naltrexone resulting in the patients' symptoms worsening and engaging in prior habits. Both patients went through periods of not receiving naltrexone, which resulted in a reversal of prior habits. Patient 1 chronically ate “massive amounts” of string to the point of surgical interventions, and patient 2 showed a decreased ability to control habits like chewing on pencils and biting her skin. Some studies indicate that ASD symptoms can also be controlled by naltrexone due to its potential underlying opioid pathway implications [20]. Our patients continue to receive psychological support via therapy and have an optimized medical regimen that shows continued benefits.

For our first patient, they noted significant improvements with the increase in fluoxetine and starting naltrexone at the same time. Although one may consider the increase in fluoxetine as a potential confounding variable to the true effect of Naltrexone, it should be noted that for a brief period, the patient was unable to obtain naltrexone, and during that time, they had significant worsening anxiety and an increase in compulsive behaviors. Their symptoms only improved again after they were able to restart the naltrexone. Similarly, in the second case report, the patient’s compulsive habit of picking on chewing greatly improved right after starting the medications, and when they skipped a dose, those symptoms returned. Although the results were stark and nearly immediate for both cases, the limitations of the study include challenges with accurately monitoring the length of time needed to see improvements from naltrexone consistently versus the length of time needed to continue the medications indefinitely or to stabilize the symptoms before discontinuing the medication. Therefore, individual case reports may not be generalizable, and further studies with an increased sample size of children and adolescents should be evaluated for the intended and adverse effects of naltrexone in these specific patient subpopulations.

## Conclusions

In conclusion, continuing the research by studying the multiple benefits of naltrexone in adult and pediatric populations would positively impact the psychiatric community. Expanding naltrexone’s purpose from the category of “medication-assisted treatment” to include additional treatment of compulsive behaviors like pica, trichotillomania, or skin excoriations would greatly help those with severe anxiety or autism. While there is still more to learn about naltrexone and its effects on pediatric populations, our report serves as a catalyst for continued exploration into the therapeutic potential of naltrexone in pediatric psychiatric clinical practice. We advocate for collaborative efforts to conduct further clinical trials aimed at further clarifying the role of naltrexone in pediatric populations and optimizing patient outcomes.

## References

[1] Luigjes J, Lorenzetti V, de Haan S (2019). Defining compulsive behavior. Neuropsychol Rev.

[2] Brock H, Hany M (2024). Obsessive-Compulsive Disorder. http://www.ncbi.nlm.nih.gov/books/NBK553162/.

[3] Al Nasser Y, Muco E, Alsaad AJ (2024). Pica. http://www.ncbi.nlm.nih.gov/books/NBK532242/.

[4] Barton JC, Barton JC, Bertoli LF (2010). Pica associated with iron deficiency or depletion: clinical and laboratory correlates in 262 non-pregnant adult outpatients. BMC Blood Disord.

[5] Pereyra AD, Saadabadi A (2024). Trichotillomania. StatPearls.

[6] Hirota T, King BH (2023). Autism spectrum disorder: a review. JAMA.

[7] Sharma SR, Gonda X, Tarazi FI (2018). Autism spectrum disorder: classification, diagnosis and therapy. Pharmacol Ther.

[8] (2024). Basics About Autism Spectrum Disorder (ASD). https://www.cdc.gov/autism/index.html.

[9] Valbrun LP, Zvonarev V (2020). The opioid system and food intake: use of opiate antagonists in treatment of binge eating disorder and abnormal eating behavior. J Clin Med Res.

[10] Gianoulakis C (2001). Influence of the endogenous opioid system on high alcohol consumption and genetic predisposition to alcoholism. J Psychiatry Neurosci.

[11] Strangio AM, Rinaldi L, Monniello G, Sisti LG, de Waure C, Janiri L (2017). The effect of abuse history on adolescent patients with feeding and eating disorders treated through psychodynamic therapy: comorbidities and outcome. Front Psychiatry.

[12] Williams DE, McAdam D (2012). Assessment, behavioral treatment, and prevention of pica: clinical guidelines and recommendations for practitioners. Res Dev Disabil.

[13] Grant JE, Potenza MN, Weinstein A, Gorelick DA (2010). Introduction to behavioral addictions. Am J Drug Alcohol Abuse.

[14] Anton RF (2008). Naltrexone for the management of alcohol dependence. N Engl J Med.

[15] Singh D, Saadabadi A (2024). Naltrexone. http://www.ncbi.nlm.nih.gov/books/NBK534811/.

[16] Stancil SL, Abdel-Rahman S, Wagner J (2021). Developmental considerations for the use of naltrexone in children and adolescents. J Pediatr Pharmacol Ther.

[17] Scaife JC, Eraifej J, Green AL, Petric B, Aziz TZ, Park RJ (2022). Deep brain stimulation of the nucleus accumbens in severe enduring anorexia nervosa: a pilot study. Front Behav Neurosci.

[18] Schnitzler E (2022). The neurology and psychopathology of Pica. Curr Neurol Neurosci Rep.

[19] Kolmen BK, Feldman HM, Handen BL, Janosky JE (1995). Naltrexone in young autistic children: a double-blind, placebo-controlled crossover study. J Am Acad Child Adolesc Psychiatry.

[20] Desjardins S, Doyen C, Contejean Y, Kaye K, Paubel P (2009). [Treatment of a serious autistic disorder in a child with Naltrexone in an oral suspension form]. Encephale.

